# Measures of Potential Flexibility and Practical Flexibility in Equation Solving

**DOI:** 10.3389/fpsyg.2017.01368

**Published:** 2017-08-10

**Authors:** Le Xu, Ru-De Liu, Jon R. Star, Jia Wang, Ying Liu, Rui Zhen

**Affiliations:** ^1^Institute of Developmental Psychology, Beijing Key Laboratory of Applied Experimental Psychology, Faculty of Psychology, Beijing Normal University Beijing, China; ^2^Graduate School of Education, Harvard University Cambridge, MA, United States

**Keywords:** strategic flexibility, potential flexibility, practical flexibility, measures, equation solving

## Abstract

Researchers interested in mathematical proficiency have recently begun to explore the development of strategic flexibility, where flexibility is defined as knowledge of multiple strategies for solving a problem and the ability to implement an innovative strategy for a given problem solving circumstance. However, anecdotal findings from this literature indicate that students do not consistently use an innovative strategy for solving a given problem, even when these same students demonstrate knowledge of innovative strategies. This distinction, sometimes framed in the psychological literature as competence vs. performance—has not been previously studied for flexibility. In order to explore the competence/performance distinction in flexibility, this study developed and validated measures for *potential* flexibility (e.g., competence, or knowledge of multiple strategies) and *practical* flexibility (e.g., performance, use of innovative strategies) for solving equations. The measures were administrated to a sample of 158 Chinese middle school students through a Tri-Phase Flexibility Assessment, in which the students were asked to solve each equation, generate additional strategies, and evaluate own multiple strategies. Confirmatory factor analysis supported a two-factor model of potential and practical flexibility. Satisfactory internal consistency was found for the measures. Additional validity evidence included the significant association with flexibility measured with the previous method. Potential flexibility and practical flexibility were found to be distinct but related. The theoretical and practical implications of the concepts and their measures of potential flexibility and practical flexibility are discussed.

## Introduction

Strategic flexibility is considered to be one important component of mathematical proficiency, where flexibility is defined as knowledge of multiple strategies and the ability to use these strategies in innovative ways in different problem solving situations (Baroody and Dowker, 2003; Rittle-Johnson and Star, 2007; Schneider et al., 2011). In the past decades, flexibility has been increasingly studied and has received considerable attention in educational practices as an important component of students' higher-order thinking ability and creativity (Verschaffel et al., 2009). Researchers (Lemaire et al., 2000; Star and Rittle-Johnson, 2008; Zhang, 2015) have found that students often did not exhibit flexibility during actual problem solving, despite having demonstrated knowledge of standard and innovative strategies on prior measures. This study aimed to develop a reliable and valid measuring method for strategic flexibility that can distinguish between what students know about standard and innovative strategies and what they actually use during problem solving. The development of this kind of measure can be taken as an important step in promoting further research that explores the reasons behind this phenomenon and instructional interventions that can improve students' flexibility.

### Distinction between competence and performance

Anecdotal findings from the previously mentioned literature indicate that students did not consistently use an innovative strategy for solving a given problem, even when these same students demonstrated knowledge of innovative strategies. For example, Star and Rittle-Johnson (2008) reported that students who were prompted during a problem-solving intervention to solve equations using multiple (e.g., standard and innovative) strategies were able to demonstrate knowledge of multiple strategies but only used more innovative strategies on 22% of posttest problems. Similarly, Star and Seifert (2006) found that students who received an intervention focusing on developing knowledge of multiple strategies used an innovative strategy on only 9% of posttest problems.

Similar results have been found within the larger literature on problem solving in the US (e.g., Carry et al., 1979), France (e.g., Lemaire et al., 2000), and China (Zhao, 2010; Zhang, 2015). Learners—even those who have demonstrated knowledge of multiple strategies—do not always choose to use innovative strategies but instead consistently rely upon standard strategies. The same is true for experts; Star and Newton (2009; see also Dowker, 1992) found that even experts did not always use innovative strategies for a given problem, despite showing explicit preferences for (and knowledge of) the innovative strategies as determined by later interviews.

Psychologists have long recognized this distinction, between knowledge of strategies (referred to as competence) and the ability to implement these strategies under appropriate circumstances (referred to as performance) (e.g., Flavell and Wohlwill, 1969; Le Corre et al., 2006; Lobina, 2011). This finding is also consistent with research on strategy learning and choice, which finds that strategy variability persists, even as learners gradually acquire problem solving expertise (Siegler and Shrager, 1984; Lemaire and Siegler, 1995; Siegler and Shipley, 1995; Siegler and Lemaire, 1997).

### Potential flexibility and practical flexibility

Extending this distinction between competence and performance into the study of flexibility, here we define *potential flexibility* as the knowledge of multiple (standard and innovative) strategies for solving mathematics problems and *practical flexibility* as the ability to implement innovative strategies for a given problem. Theoretical support for the distinction between potential and practical flexibility—in addition to the above-mentioned work of Star and colleagues (e.g., Star and Seifert, 2006; Star and Rittle-Johnson, 2008) comes from multiple sources.

In particular, Frick et al. (1959) found two distinct factors of flexibility of thinking: spontaneous flexibility and adaptive flexibility. Spontaneous flexibility was defined as “the ability to generate a diversity of ideas in relatively unstructured situation” (Frick et al., 1959, p. 471), while adaptive flexibility was defined as “the ability to change set in order to meet requirements by changing problems” (Frick et al., 1959, p. 471). This prior study provides support from the literature that flexibility can be classified into different subtypes. Furthermore, our construct of potential flexibility appears to be somewhat similar to “spontaneous flexibility”—the ability to produce multiple ideas, while practical flexibility seems somewhat similar to “adaptive flexibility”—the ability to generate innovative ideas for changeable problem solving situations.

Also, Verschaffel et al. (2009, pp. 337–338) made a distinction that appears similar to the one in this study between potential flexibility and practical flexibility. They used “flexibility” to refer to knowledge of multiple strategies; “adaptivity” to refer to the ability to use innovative strategies for a given problem; and “flexibility/adaptivity” to refer to the overall construct which combines these two. Accordingly, our construct of potential flexibility appears to be similar to Verschaffel and colleagues' “flexibility,” while practical flexibility seems similar to “adaptivity.” However, our conceptualization of potential and practical flexibility differs from this prior work, primarily in how we assess flexibility. For Verschaffel et al. (2009), flexibility and adaptivity are two distinct components of (what they call) flexibility/adaptivity, assessed by identical tasks. In contrast, we view potential and practical flexibility as two different types of flexibility that are potentially elicited by two different kinds of tasks (described in more depth below).

Thus the literature on strategy flexibility suggests that there is a distinction between potential flexibility and practical flexibility. The former is focused on students' ability to generate multiple (standard and innovative) strategies, while the latter involves the performance or use of innovative strategies.

### Assessment of flexibility in mathematics

As noted above, prior research on flexibility has anecdotally reported on students' deficiencies in practical flexibility (e.g., Star and Seifert, 2006; Star and Rittle-Johnson, 2008). Yet within this literature, researchers have not examined the relationship between the practical and potential flexibility nor have they sought explanations for why individuals might have different degrees of potential and practical flexibility. Examining this relationship requires the development of ways to reliably measure potential and practical flexibility.

The development of our measure for potential and practical flexibility was informed by the ways that prior researchers have measured flexibility, particularly how existing work has tried to distinguish between knowledge of multiple strategies and the ability to use innovative strategies. As we describe below, there seems to be some indecision among researchers who study flexibility as to whether this construct is best measured via processes of recognition and evaluation, via processes of generation, or some combination of the two.

Many studies infer flexibility from students' ability to recognize and evaluate multiple and innovative strategies. Students are provided with examples of problem solving strategies and asked to indicate (often via multiple choice questions) whether these strategies are legitimate and/or innovative ways to solve problems. For example, in Rittle-Johnson and Star (2007), students were given the equation 2(*x* + 1) + 4 = 12 and asked, in a multiple choice question, to identify all possible steps that could be done next. Students' ability to identify multiple possible next steps was interpreted to indicate knowledge of multiple strategies. Similarly, the flexibility measure in Star et al. (2015) included a multiple choice item asking students to select the innovative first step for solving a given equation—where responses were taken to indicate knowledge of innovative strategies.

Other studies rely more on processes of strategy generation for measuring flexibility. Students are asked to solve problems (often more than once), and analyses of the strategies that they generate (e.g., whether students are able to generate multiple strategies and/or innovative strategies) are used to infer flexibility. An early example of this approach was utilized by Krutetskii (1976), who directly asked students to solve problems several times using multiple strategies. Van der Heijden et al. (1993, as cited in Verschaffel et al., 2009) followed a similar approach, inferring flexibility from whether students used multiple and innovative strategies for solving mental addition and subtraction problems. Related, Blöte et al. (2001) used “the flexibility-on-demand task” (FDT; Klein, 1998), where students were asked to re-solve previously completed problems but using a different strategy. Star and Seifert (2006) used a variant of this task which they referred to as the “alternative ordering task.” After solving each problem twice, students were asked to select the innovative strategy for each problem from among the strategies that they had generated.

Other studies include a mix of recognition/evaluation and generation items to try to better capture both students' knowledge of multiple strategies as well as their ability to use innovative strategies. For example, Star and Rittle-Johnson (2008) explicitly distinguish between what they refer to as flexibility knowledge and flexibility use. Within flexibility knowledge, there are items that tap knowledge of multiple strategies (e.g., accepting multiple solution strategies, identifying multiple next steps) and knowledge of innovative strategies (recognition and evaluation of innovative steps). For flexibility use, Star and Rittle-Johnson analyzed students' strategies on post-test problems to code for whether students used multiple strategies and/or used innovative strategies.

Another way that researchers have attempted to address the dual challenges of assessing what strategies students know as well as whether they can successfully implement these strategies is through the choice/no-choice method. This method was first introduced by Siegler and Lemaire (1997) but has been widely used by many subsequent researchers (e.g., Torbeyns et al., 2006, 2009; Luwel et al., 2009; Lemaire and Lecacheur, 2010; Torbeyns and Verschaffel, 2013). The choice/no-choice method involves presenting students with problems to solve in a “choice” condition, where students are allowed to choose which strategy to use for a given problem, and a “no-choice” condition, where students are told which strategy they must use. Through analyses of speed and accuracy in both conditions, as well as strategy usage in the choice condition, has been used to tap students' knowledge of strategies as well as their ability to use innovative strategies.

Our approach to measuring potential and practical flexibility drew from all of the work described above and represents an awareness of the challenges of adequately measuring both what students know and also what the strategies that they can actually use. For example, recognition and evaluation can be efficient approaches to measuring flexibility, as it can be very time-consuming for students to have to generate multiple strategies for many problems. Yet it is much easier to show recognition of multiple and innovative strategies than to generate these strategies (e.g., Hollingworth, 1913); reliance on recognition could result in over-estimates of students' ability to actually implement strategies. At the same time, relying exclusively on generation methods can result in underestimates of flexibility. As noted earlier, students' ability to implement known strategies often lags substantially behind their knowledge of these same strategies. Our assessment attempted to overcome this challenge by combining generation and evaluation methods.

### Framework for measuring potential flexibility and practical flexibility

In order to assess potential and practical flexibility, our measure (described in more depth below) asked students to go through the same set of problems three times; therefore we refer to it as a Tri-Phase Flexibility Assessment. In Phase One, students were asked to solve each problem as quickly and accurately as possible, which was intended to prompt students to select and implement an innovative problem-solving strategy. This gave us a generation-based measure of students' default/preferred strategy for each problem. In Phase Two, students were asked to go through the assessment again and generate multiple strategies for each problem in addition to the strategy they produced in Phase One. In Phase Three, students were asked to evaluate their own strategies generated in the former two phases for each problem, selecting the innovative one.

Based on the definitions of potential and practical flexibility mentioned above, students' flexibility was evaluated using their responses to each problem in the three phases. Practical flexibility was evaluated based on whether the strategy that was produced for each problem in Phase One was innovative. If a student used an innovative strategy for a given problem (where “innovative” was operationalized as the strategy for that problem that had the fewest steps and with the most simplified computations, consistent with prior research e.g., Star and Rittle-Johnson, 2008; Heinze et al., 2009; Star and Newton, 2009), he or she would be assessed as having practical flexibility for that problem. Potential flexibility was evaluated based on the combination of the variety of strategies students generated in Phases One and Two and the innovativeness of the strategy that was selected in Phase Three. If a student generated multiple (standard and innovative) strategies for a given problem and then recognized the innovative one from among them, he or she would be assessed as having potential flexibility for that problem.

### The present study

The aim of this study was to develop reliable and valid measures of potential and practical flexibility, using the domain of algebra equation solving as the focus of investigation. Algebra was chosen for several reasons. First, algebra is considered by many to be students' first sustained exposure to the abstraction that makes mathematics powerful (Fey, 1990; Kieran, 1992). Equation solving is a core yet challenging component of algebra (Blume and Heckman, 1997; Schmidt et al., 1999). Furthermore, equation solving is a subdomain of algebra where flexibility in the use of strategies seems particularly useful and as a result has been frequently studied (e.g., Star and Seifert, 2006; Rittle-Johnson and Star, 2007; Star et al., 2015).

As described below, we developed measures of potential and practical flexibility and then administrated them to seventh grade Chinese students. We performed psychometric analyses to provide measurement indicators, such as internal consistency, factorial validity and criterion-related validity to validate our measures of potential and practical flexibility in equation solving.

## Method

### Participants

The 158 seventh grade participants (93 female, 65 male; ages ranged from 11 to 14 years, *M* = 12.74, *SD* = 0.56) in this study were recruited from six classrooms within the same region and school system in a northern city of China. By analyzing students' scores of the latest school-level mathematics test, the ANOVA analysis revealed that there were no differences in these six classrooms in terms of students' average mathematics achievement [*F*_(5, 152)_ = 0.16, *p* = 0.97 > 0.05].

At the time of this study, all participants learned from the same curriculum materials, as all teachers reported closely adhering to the Mathematics Curriculum Standards in China. In particular, prior to the start of the study, students had been taught both a standard algorithm for solving linear equations as well as more innovative strategies for how to solve equations. The standard algorithm taught by teachers was the same algorithm referred to in the literature (e.g., Star and Seifert, 2006)—first, expand the parentheses, then combine terms, then subtract from both sides, and finally divide to both sides. Innovative strategies involved combining these four steps in atypical sequences. During lessons on solving linear equations prior to the study, students were at times asked to solve equations using both standard and innovative methods. Also, conversations with the teachers of these seventh grade classes indicated that the use of multiple solution methods was sometimes encouraged in their mathematics classes.

### Ethical statement

The study was approved by the ethical committee of the School of Psychology at Beijing Normal University. Written informed consents were obtained from the schools, teachers, parents, and all participants prior to initiating the study. All participants were informed that they had the right to withdraw from this study at any time.

### Assessment

The assessment contained 12 linear equations (see **Appendix** for a list of all problems solved by students during the problem-solving sessions). These problems were designed so that each could be solved using a standard algorithm but where a more innovative strategy also existed.

The 12 problems were divided into four problem types, with three instances of each problem type (see Table 1). The first three problem types were identical to those used in prior work (e.g., Star and Rittle-Johnson, 2008); the fourth problem type was new. Problem One to Problem Three (type: DIVIDE) were of the form a(*x*–b) = c, where c was evenly divisible by a. The innovative strategy involved dividing both sides of the equation by a as a first step. Problem Four to Problem Six (type: COMBINE) were of the form a(*x* + c) + b(*x* + c) = d. The innovative strategy involved adding together the (*x* + c) terms as a first step. Problem Seven to Problem 10 (type: SUBTRACT FROM BOTH) were of the form a(*x* + c) = b(*x* + c) + d, where the innovative strategy involved collecting the (*x* + c) terms together as a first step. Finally, problems 10–12 (type: SIMPLIFY FRACTION) included fractions, where the innovative strategy involving either multiplying both sides by a constant to “clear” fractions as a first step or simplifying fractions as a first step.

**Table 1:** Types of shortcuts of example equations attempted on the problem-solving session.

**Innovational transformation**	**Example problem**	**Solution using standard algorithm**	**Solution using innovative strategy**
DIVIDE	3(*x* − 1) = 27	3*x* − 3 = 27 3*x* = 30 *x* = 10	*x* − 1 = 9 *x* = 10
COMBINE	2(*x* + 3) + 3(*x* + 3) = 20	2*x* + 6 + 3*x* + 9 = 20 5*x* + 15 = 20 5*x* = 15 *x* = 1	5(*x* + 3) = 20 *x* + 3 = 4 *x* = 1
SUBTRACT FROM BOTH	$5\left(x-\frac{2}{3}\right)-10=2\left(x-\frac{2}{3}\right)$	$\begin{array}{cc} 5x-\frac{10}{3}-10= & 2x-\frac{4}{3}\\ 5x-2x= & \frac{10}{3}+10-\frac{4}{3}\\ 3x= & 12\\ x= & 4 \end{array}$	$\begin{array}{cc} 3\left(x-\frac{2}{3}\right)= & 10\\ x-\frac{2}{3}= & \frac{10}{3}\\ x= & 4 \end{array}$
	4(*x* + 2.5) + 5*x* = 4(*x* + 2.5) + 8	$\begin{array}{cc} 4x+10+5x= & 4x+10+8\\ 9x+10= & 4x+18\\ 9x-4x= & 18-10\\ 5x= & 8\\ x= & \frac{8}{5} \end{array}$	$\begin{array}{cc} 5x= & 8\\ x= & \frac{8}{5} \end{array}$
SIMPLIFY FRACTION	$\frac{3x+3}{3}+\frac{4x+4}{4}=4$	$\begin{array}{cc} 12\times \left(\frac{3x+3}{3}+\frac{4x+4}{4}\right)= & 12\times 4\\ 12x+12+12x+12= & 48\\ 24x+24= & 48\\ 24x= & 24\\ x= & 1 \end{array}$	*x* + 1 + *x* + 1 = 4 *x* + 1 = 2 *x* = 1

Note that in a departure from prior work that used similar problems (e.g., Star and Rittle-Johnson, 2008), we included problems that had fractions as well as decimals, which likely increased the arithmetic complexity and difficulty level of the problems for students. Our decision to make these problems more difficult was driven by the following reasons. First, we sought to minimize the possibility of “routinization” (Spiro, 1980), which might come up when participants faced isomorphic problems with integral coefficients and constants. Second, we felt that the use of harder problems allowed us to more accurately assess the full range of students' mathematical knowledge about equation solving. Third, prior work has suggested that students may be more likely to select innovative strategies when facing more difficult problems (Newton et al., 2010), such as those containing fractions and decimals.

### Procedure

All students worked individually on the 12 problems on the assessment in one 90-min session that was conducted during students' regular mathematics classes. In addition to the three phases of Tri-Phase Flexibility Assessment, we designed a fourth phase. In the fourth phase, students were asked to evaluate a list of strategies provided by experts for each problem, selecting the innovative ones. Data from the fourth phase was regarded as the criterion for potential flexibility. Thus, the study procedure included four phases (see Table 2).

**Table 2:** Overview of Procedure (time unit: minute).

**Phase**	**Duration**	**Activity**
1	20	Solving all 12 problems as accurately and quickly as possible.
2	30	Generating additional strategies for all 12 problems.
3	20	Evaluating one's own strategies and selecting the one felt to be innovative for each problem.
4	20	Evaluates the strategies from a Strategy Key and selecting the one felt to be innovative for each problem.

In Phase One, each student attempted to solve each of the 12 problems, working at their own pace. Below each problem was a large square where students could write their strategy and solution. The experimenter instructed participants to solve all problems as accurately and quickly as possible and to show all of their intermediate steps. Students were required to solve the problems in numerical order. If students finished all of these problems before time had been called, they were instructed to close their test booklets and sit quietly. Phase One had two goals: (a) to determine if students could correctly solve each equation, and (b) to investigate whether students used an innovative strategy to solve each problem (e.g., practical flexibility).

In Phase Two, students were asked to begin the test all over again with the first problem and re-solve each problem, using as many different strategies as they could think of. In addition to the box below each problem containing the students' first (Phase One) strategy, there were also five additional boxes where students could write alternative strategies. Students were not allowed to change or add to their Phase One; students were required to work through the problems in numerical order. The aim of Phase Two was to determine whether each student had knowledge of multiple strategies for each problem.

In Phase Three, students were instructed to look over all of the strategies that they had generated for each problem in Phase One and Two and to select the strategy that they felt was the innovative one for each problem (by placing a check mark by the selected strategy). Students were not allowed to change any of their solutions from Phase One or Two during Phase Three. The aim of Phase Three was to determine whether students had knowledge of innovative strategies. After Phase Three was completed, students handed in their tests to the experimenter.

Finally, in Phase Four, students were handed a Strategy Key to the test. The Strategy Key listed several correct strategies for each problem, including the standard algorithm, an innovative strategy for each type, and other strategies that were neither standard nor innovative. The strategies for each problem were listed in random order. Students were asked to select the innovative strategy for each problem from the strategies shown on the Strategy Key. On the Strategy Key, we attended carefully to the number of lines/steps in each strategy, in order to prevent participants from identifying the innovative strategy merely by selecting the one with the fewest lines. The goal of Phase Four was the same as in Phase Three.

### Coding

Students' work from all four Phases was coded by three independent coders, all of whom were doctoral students in mathematics education. At least two coders looked at each dimension of coding (described below). When disagreements arose, the third rater contributed her coding, and the three coders met to resolve the disagreement.

Recall that our interest is in the measurement of two types of flexibility: potential flexibility and practical flexibility. Practical flexibility was operationally defined as follows. If a learner was able to implement an innovative strategy for solving a given equation in his/her first attempt at solving the problem, the learner was said to have a high level of practical flexibility for that problem. A learner who was only able to implement a standard approach in his/her first attempt at solving the problem was judged to have low levels of practical flexibility. Thus, practical flexibility is about spontaneously putting one's knowledge of strategies in action. Potential flexibility was operationally defined as follows. If a learner demonstrated knowledge of both standard and innovative strategies for a given problem, he/she was said to have high potential flexibility. If a learner could not produce multiple (e.g., standard and innovative) strategies, he/she was said to have low potential flexibility.

Determining scores for potential and practical flexibility required coding for the following constructs.

### Strategy generation

A Strategy Generation score indicated the extent that students knew multiple strategies for solving a given problem. A coding scheme was developed for each problem to determine whether students knew the standard strategy and also whether students knew an innovative strategy for that problem. Coders looked at students' work across Phase One and Two of each problem. Students' strategies were coded into one of three categories—standard strategy, innovative strategy or other. For this coding, computational errors were ignored. As described above, the standard strategy was defined as first distributing, then combining like terms (if possible), then adding/subtracting from both sides, and finally dividing/multiplying on both sides (Star and Seifert, 2006). The innovative strategy was made use of an innovative first step (for the DIVIDE type, dividing first; for the COMBINE type, combining first; see above). All other attempted solution methods were coded as “other,” and these included use of other nonconventional strategies, strategies that violated mathematical principles, and incomplete strategies that were too ambiguous to code as either standard or innovative use. If a student demonstrated knowledge of both standard and innovative strategies, the student received a Strategy Generation score of one for that problem. Otherwise, the student received a Strategy Generation score of zero. Given that each student received a Strategy Generation score for every problem, the maximum score of Strategy Generation was 12. The interrater reliability for the strategy generation scores was 0.95.

Note that the distinction between a standard strategy and an innovative strategy could usually be determined by analyzing the first one or two steps in each method. For example, the standard strategy for Item One to Item Nine began by distributing the parentheses, and the standard algorithm for items 10–12 involved obtaining a common denominator for the two algebraic expressions and then combining the two expressions. Similarly, from Item One to Item Three, the innovative strategy involved dividing a constant to both sides before distributing (DIVIDE); from Item Four to Item Six, the innovative strategy involved combining same terms on one side (COMBINE); from Item Seven to Item Nine, the innovative strategy involved subtracting same terms from both sides (SUBTRACT FROM BOTH); from Item 10 to Item 12, the innovative strategy involved first reducing each fraction before combining (SIMPLIFY FRACTION) (see Table 1; see also **Appendix** for a list of all problems solved by students during the problem-solving sessions).

### Strategy evaluation

The Strategy Evaluation score indicated whether students were able to identify the innovative strategy for each problem, from among the methods that were student-generated. Raters looked at each student's strategies for each problem (from Phase One and Two) and identified one or more that were innovative. Then, for each problem, if students selected (in Phase Three) a strategy that raters identified as innovative, one point was earned. If students selected a different non-innovative strategy, no points were earned for that problem. The maximum score of Strategy Evaluation was 12. The interrater reliability for strategy evaluation was scores 0.96.

### Potential flexibility

Potential Flexibility was a composite score indicating whether students knew multiple strategies and were able to identify the innovative one among strategies that they knew. In particular, the Potential Flexibility score for each problem was determined from the Strategy Generation score and the Strategy Evaluation score. For a given problem, if Strategy Generation was one point (indicating that students produced both standard and innovative strategies for that problem) and Strategy Evaluation was one point (indicating that students were able to identify which strategy for that problem was innovative), students earned a Potential Flexibility score of one. Otherwise, the Potential Flexibility score was zero for that problem. The maximum score of the Potential Flexibility was 12. The interrater reliability for potential flexibility scores was 0.97.

### Practical flexibility

Practical Flexibility measures whether students are able to use an innovative strategy on their first attempt at solving a problem. For each problem, raters analyzed each student's first solution attempt (from Phase One) to determine whether this strategy was innovative or not. If the first strategy was determined to be innovative, the student earned a Practical Flexibility score of one. Otherwise, the Practical Flexibility score was zero for that problem. The maximum score of the Practical Flexibility was 12. The interrater reliability for practical flexibility scores was 0.97.

### Accuracy score

In addition to coding for potential and practical flexibility, the accuracy of each participant's solutions was also coded.

The accuracy score indicated whether students were able to correctly solve given equations (arriving at the correct numerical answers) on the first attempt. Students were given one point for each problem where a correct answer was generated and zero points for incorrect answers. The maximum score of Accuracy was 12. The interrater reliability for accuracy scores was 0.98.

### Strategy identification

As a criterion for potential flexibility, strategy identification (students' selections from the Strategy Key) in Phase Four was also coded. The Strategy Identification score indicated whether students were able to recognize the innovative strategy for each problem, from among the methods that were seen in the Strategy Key. Expert trained raters looked at each strategy in the key and identified one or more that were innovative for a given problem. For each problem, if students selected a strategy that raters identified as innovative, one point was earned. If students selected a different non-innovative strategy, no points were earned for that problem. The maximum score of Strategy Identification was 12. The interrater reliability for strategy identification scores was 0.96.

## Results

### Descriptive statistics of all variables

General means and standard deviations for all variables are depicted in Table 3.

**Table 3:** Descriptive statistics of all variables (*N* = 158).

**Variables**	***M***	***SD***
Accuracy	10.66	1.58
Strategy Generation	7.09	4.11
Strategy Evaluation	6.23	4.22
Strategy Identification	8.30	2.83
Potential Flexibility	5.85	4.15
Practical Flexibility	1.44	2.60

Accuracy on equation solving was relatively high (*M* = 10.66) with a rate of 88.83%, indicating the fact that the vast majority of participants were able to correctly solve most of the items. Participants showed moderate levels of potential flexibility and comparatively low levels of practical flexibility. A paired-samples *T*-test showed that participants earned significantly higher potential flexibility scores than practical flexibility scores (*t* = 12.97, *p* < 0.001).

### Distribution of scoring percentage on each item

**Figure 1:**
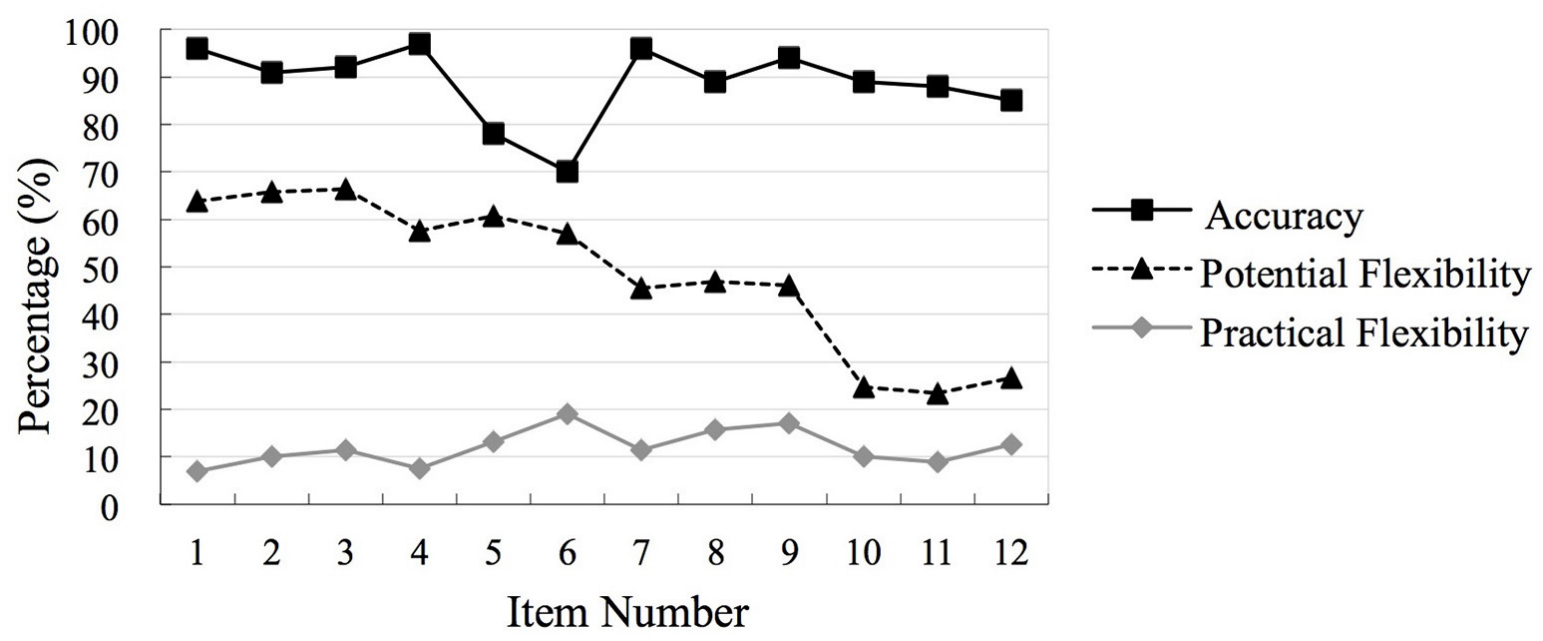
Distribution scoring percentage of accuracy. Practical flexibility and practical flexibility on each item (*N* = 158).

The distribution of scoring percentage of accuracy, potential flexibility and practical flexibility on each item is presented in Figure 1.

As seen in Figure 1, on DIVIDE items, more than 63% of participants had Potential Flexibility scores of one on each problem. This percentage declined to 57% for COMBINE items, 46% on SUBTRACT FROM BOTH SIDES items, and 25% on SIMPLIFY FRACTION items. This suggests that the DIVIDE strategy was the easiest one to generate and identify, followed by the COMBINE and SUBTRACT FROM BOTH SIDES strategies, and with the SIMPLIFY FRACTION strategy as the most difficult one for participants to implement. The findings confirmed that the assessment had a good structure because from Item One to Item 12, the difficulty of equation problems increased.

Interestingly, the results of the frequency distribution of participants' scores on each item for Practical Flexibility were different from that for Potential Flexibility. Recall that Practical Flexibility score of one resulted when a student's first attempt on a problem used an innovative strategy. As displayed in Figure 1, on each item <19% of participants got one point for Practical Flexibility.

Participants' accuracy scores were generally higher than both potential and practical flexibility scores. Further frequency analysis (see Table 4) showed that in 40% of cases, students correctly solved an equation but did not demonstrate potential flexibility or practical flexibility, whereas in 38% of cases, students correctly solved an equation and demonstrated potential flexibility, but did not show practical flexibility. Only in 8% of cases did students correctly solve an equation and demonstrate both potential and practical flexibility.

**Table 4:** Percentage of scoring combinations in accuracy, potential and practical flexibility (*N* = 158).

**Combination**	**Accuracy**	**Potential flex**.	**Practical flex**.	**Percentage (%)**
None Scoring	0	0	0	8
Only Accuracy	1	0	0	40
No Practical Flex.	1	1	0	38
All Scoring	1	1	1	8
Others				6

### Internal consistency of measures

The internal consistency coefficient (Cronbach's alpha) for Potential Flexibility was found to be 0.92, and the internal consistency coefficient (Cronbach's alpha) of Practical Flexibility was 0.89. Values of Cronbach's alpha that are above 0.70 are considered to be acceptable (Nunnally, 1978).

### Confirmatory factor analysis

**Figure 2:**
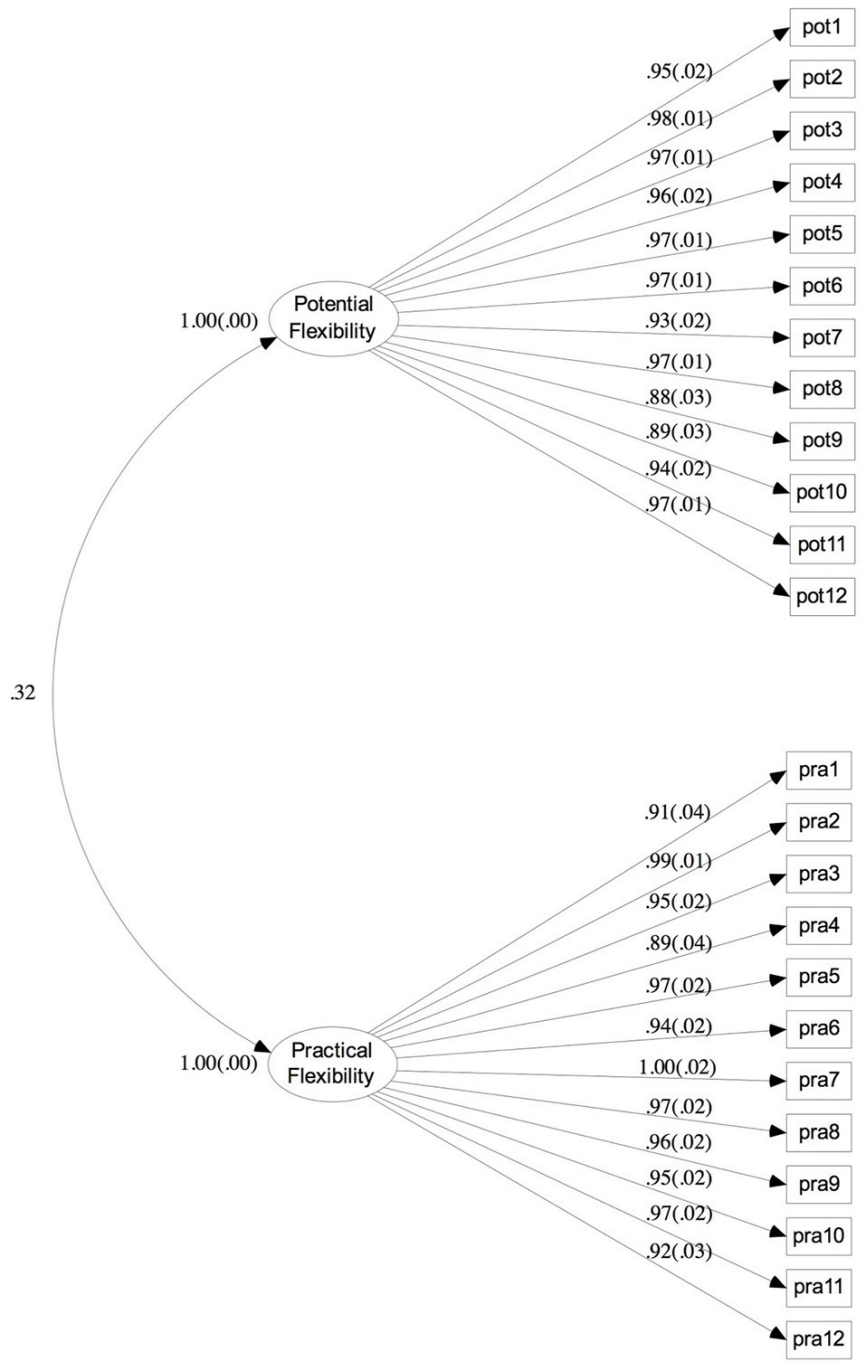
Factor loading and factor intercorrelations for potential flexibility and practical flexibility. All estimated coefficients are significant with *p* < 0.001. Pot, Potential Flexibility Item; Pra, Practical Flexibility Item.

We conducted a Confirmatory Factor Analysis (CFA) to examine whether the estimated model (shown as Figure 2) fit well with the current data set. Considering that the type of observed variables were categorical factors and the sample size here was <200, we utilized Weight Least Square with Mean and Variance (WLSMV; Muthén, 1993; Muthén et al., unpublished manuscript) to estimate the path coefficients (Flora and Curran, 2004; Beauducel and Herzberg, 2006). The overall fitting indexes of the model were as the following: χ ^2^*/df* = 51.75 (*p* < 0.001), WRMR = 1.86, TLI = 0.98, CFI = 0.98, RMSEA = 0.09. Values > 0.90 for both the TLI and CFI suggest plausible model fit for the data, and values > 0.95 for both of them indicate good model fit (Hu and Bentler, 1995; Hair et al., 1998). In addition, values < 0.1 for RMSEA suggest acceptable model fit (Steiger, 1990). The analysis results indicated a good fit of model.

### Criterion-related validity

Strategy identification was a method used to assess flexibility in prior studies (e.g., Star et al., 2015). Because of the similarity between these measures, Strategy Identification was regarded as a criterion for potential flexibility in our study. As mentioned above, strategy identification scores indicated whether students had knowledge of the innovative strategy for each problem. Therefore, high strategy identification was expected to have a strong correlation with high potential flexibility. The criterion-related validity was tested. The potential flexibility was positively correlated with Strategy Identification (*r* = 0.38, *p* < 0.01).

### Composite reliability and convergent validity

Furthermore, we tested composite reliability (CR) and convergent validity of potential flexibility and practical flexibility (see Table 5). Bagozzi and Yi (1988) suggested two standards for testing the reliability of scales: (a) the path coefficients between observed variables and latent variables should be significant and the square of each path coefficient should be > 0.20 (Jöreskog and Sörbom, 1989; Bentler and Wu, 1993); (b) the composite reliability (CR) values of latent variables should be >0.60 (Fornell and Larcker, 1981). With regard to estimating convergent validity, two aspects should be taken into account: (a) the path coefficients between observed variables and latent variables should be significant and their values should be >0.45 (Bentler and Wu, 1993); (b) the average variance extracted (AVE) of latent variables should be >0.50. As Table 5 shows, the measures in this study had good composite reliability and convergent validity.

**Table 5:** Composite reliability and convergent validity of potential flexibility and practical flexibility (*N* = 158).

**Latent variables**	**Observed variables**	**Standardized path coefficients**	***R^2^***	**CR**	**AVE**
Potential flexibility				0.998	0.98
	Pot 1	0.95^***^	0.90		
	Pot 2	0.98^***^	0.96		
	Pot 3	0.97^***^	0.94		
	Pot 4	0.96^***^	0.92		
	Pot 5	0.97^***^	0.94		
	Pot 6	0.97^***^	0.94		
	Pot 7	0.93^***^	0.86		
	Pot 8	0.97^***^	0.94		
	Pot 9	0.88^***^	0.77		
	Pot 10	0.89^***^	0.79		
	Pot 11	0.94^***^	0.88		
	Pot 12	0.97^***^	0.94		
Practical flexibility				0.998	0.98
	Pra 1	0.91^***^	0.83		
	Pra 2	0.99^***^	0.98		
	Pra 3	0.95^***^	0.90		
	Pra 4	0.89^***^	0.79		
	Pra 5	0.97^***^	0.94		
	Pra 6	0.94^***^	0.88		
	Pra 7	1.00^***^	1.00		
	Pra 8	0.97^***^	0.94		
	Pra 9	0.96^***^	0.92		
	Pra 10	0.95^***^	0.90		
	Pra 11	0.97^***^	0.94		
	Pra 12	0.92^***^	0.85		

*** *p < 0.001. Pot, Potential Flexibility; Pra, Practical Flexibility; R^2^, square of Standardized path coefficients; CR, Composite Reliability; AVE, Average Variance Extracted*.

### Correlation analysis

Practical flexibility was found to be positively correlated with potential flexibility (*r* = 0.27, *p* < 0.01). This result was consistent with our expectation.

## Discussion

Researchers in mathematics education are devoting increasing attention on how to develop students' ability to solve problems flexibly (Verschaffel and De Corte, 1996; Baroody and Dowker, 2003; Hatano, 2003; Siegler and Booth, 2005). The present study explored different aspects of students' strategy flexibility and developed and validated an assessment tool for evaluating students' flexibility. The results demonstrated satisfactory reliabilities, factorial validity and criterion-related validity of the measures of potential and practical flexibility in equation solving through the Tri-Phase Flexibility Assessment. Findings also indicated that the assessment was constructed well, in that the difficulty gradually increased from the DIVIDE items to the SIMPLIFY FRACTION items. Consistent with our hypothesis, our results indicated that students had significantly higher levels of potential flexibility as compared to practical flexibility, and more, potential flexibility was significantly correlated with practical flexibility. This study has theoretical and practical implications for measure development, practice and future research on mathematical flexibility.

### Measure of potential and practical flexibility

Our flexibility assessment was found to have sufficiently high reliability and acceptable validity—the former as a result of high internal consistency, and the latter demonstrated from the confirmatory factor analysis (CFA), item factor loads, criterion-related validity, composite reliability and convergent validity, all within acceptable limits. Thus, one contribution of this work is the creation of an assessment that is a promising and acceptable measurement tool for assessing potential and practical flexibility within the domain of linear equation solving.

The results showed that the accuracy scores for students on all equation solving items were much higher than the scores of both potential flexibility and practical flexibility. The results shown in Table 4 indicated that the relatively lower scores for practical flexibility were not primarily caused by students' inability to correctly solve these linear equations, as accuracy scores were quite high. Rather, flexibility scores were lower than accuracy scores because students were not able to use innovative strategies in their problem solving.

Students' lack of practical flexibility (despite high accuracy scores) may have emerged due to two possible situations. First, some students did not know multiple (standard and innovative) strategies for some problems. This situation, where students lacked both potential and practical flexibility but still were able to provide accurate solutions to many of the equations, comprised 40% of all cases. In the second situation (38% of students), students were able to accurately solve most equations and also had knowledge of standard and innovative strategies, but they were unable to use the innovative strategy as their first attempt when provided the opportunity to do so.

These findings again confirmed the phenomenon described in the introduction of this paper—that students often do not exhibit flexibility during actual problem solving, even when they have demonstrated knowledge of standard and innovative strategies on other measures. This finding not only provides support for the distinction between potential and practical flexibility but also confirms that need for measures of these constructs, to enable future research in this area.

Furthermore, the practical implications of this finding are significant. For teachers, it may be the case that there is tension between the instructional goal of helping students to answer as many problems correctly as possible, and the instructional goal of having students use innovative strategies as frequently as possible. The ability to solve problems correctly is certainly a critical goal of mathematical instruction. But a correct solution could be generated by the mere application of rote knowledge of procedures (e.g., Schmidt et al., 1999). Such rote knowledge can be resistant to transfer and thus is inflexible (Anderson and Lebiere, 1998). In contrast, the ability to select innovative strategies among diverse methods to solve problems, namely practical flexibility, can help students save time and mental effort when solving mathematical tasks and also is an indicator of deeper mathematical understanding (e.g., Blöte et al., 2001; Star and Newton, 2009). Thus, this study leaves some open questions about how to reform learning and teaching to both effectively promote the development of practical flexibility and also to enable students to be able to solve problems correctly.

### Relations between potential and practical flexibility

This study found that potential flexibility was significantly higher than and was significantly correlated with practical flexibility. This result confirmed our hypotheses that these two types of flexibility are distinct but related, and it was consistent with the findings from the psychological literature within the realm of flexibility about the relationship between potential and practical flexibility. It indicated that our measure of these two types of flexibility in equation solving through the Tri-Phase Flexibility Assessment was promising.

In addition, this finding has several implications for both theory and practice. First, we found that potential flexibility was greater than practical flexibility. Students had moderate levels of potential flexibility but comparatively low levels of practical flexibility. Many participants had demonstrated knowledge of multiple strategies, but only a few students actually executed the innovative strategy to solve equation problems in the first attempt. We interpreted this result to suggest that knowledge of problem solving strategies tends to precede the ability to innovatively implement these strategies. This finding is consistent with the literature in psychology on the distinction between competence and performance (e.g., Flavell and Wohlwill, 1969; Le Corre et al., 2006; Lobina, 2011), which indicates that learners have greater competence than they are able to innovatively implement during problem solving. In addition, this finding is also consistent with research on strategy learning, such as the Model of Strategy Change (Lemaire and Siegler, 1995), which suggests that the ability to flexibly implement new strategies is formed in later stages of strategy development. Teachers interested in promoting flexibility may need to be aware that students may have substantially greater (potential) flexibility than is evident from assessments designed to evoke practical flexibility.

Second, we found that potential flexibility was significantly correlated with practical flexibility. If an instructional goal is to develop practical flexibility—or the ability to consistently implement innovative problem solving strategies—teachers might hypothesize that the best means toward achieving this aim would be to merely provide students with instruction on only the innovative strategies. However, our results (see also Star and Rittle-Johnson, 2008) suggested that potential flexibility—knowledge of multiple strategies—was in fact an alternative route toward the achievement of practical flexibility. Providing students with knowledge of a diverse array of strategies can allow students to effectively develop the ability to use innovative strategies on a variety of problems.

Finally, one interesting finding that merits further exploration concerns the ways that, on average, potential and practical flexibility changed from the (easier) items at the beginning of the assessment to the (harder) items at the end of the assessment. We found that practical flexibility scores remained consistent (and low) throughout the assessment, despite the increasing difficulty of the problems. In contrast, potential flexibility was relatively high for the problems at the beginning of the test (averaging 63% in the first group of problems) but subsequently dropped to an average of 25% for the last group of problems. Thus, it appears that problem difficulty impacts potential and practical flexibility differently. Related, although the correlation results indicated that potential flexibility was significantly correlated with practical flexibility, the correlation coefficient between them was only 0.27. Taken together, these results indicate that there could be some other factors that influence both potential and practical flexibility. One promising candidate is students' beliefs or dispositions; future research should also consider the role of dispositional variables in the development of flexibility (Verschaffel et al., 2007, 2009). It may be the case that students' beliefs, attitudes, and habits of mind could impact (positively or negatively) their flexibility in mathematics.

### Limitations and future directions

In closing, we identify implications for future research that emerge from this study and its limitations. First, we did not conduct interviews to ask students to explain the thinking behind their strategy choices. More fine-grained qualitative information would be a very important and helpful next step to continue to advance our understanding of potential and practical flexibility. For example, why did students persist in using standard strategies to solve problems even when they knew innovative strategies? What criteria did students use to determine which strategy was innovative for a given problem?

Second, this study did not assess students' conceptual knowledge, which has been found to be distinct from but related to both flexibility and procedural knowledge (Rittle-Johnson and Star, 2009; Rittle-Johnson et al., 2009; Star and Rittle-Johnson, 2009; Schneider et al., 2011).

Third, to verify the validity of our measure, we tested criterion-related validity (strategy identification was regarded as a criterion for potential flexibility), factorial validity, composite reliability and convergent validity of our assessments. But these indicators were insufficient to definitely conclude that this test was valid. Future research can test concurrent validity and discriminant validity to further explore the validity of our measures.

Fourth, future research can adapt and refine the assessment and protocol used here for studying practical and potential flexibility in other mathematical domains. Here we created a new technique for assessing potential and practical flexibility by having students complete several passes through a set of problems in order to both generate multiple strategies as well as demonstrate knowledge of innovative strategies. This approach yielded a promising and reliable assessment for studying flexibility in linear equations. Additional work is necessary to continue to explore this form of assessment, including examining the relationship between students' performance on the various phases of the assessment. We hope that future work can verify the utility of this assessment technique in other mathematical domains, expanding our understanding of mathematical flexibility more generally.

Finally, the development of a reliable and promising measure of potential flexibility and practical flexibility in this study offers a measuring tool for future research to empirically confirm some possible theoretical explanations of the distinction between potential and practical flexibility. There were some plausible theoretical explanations from the literature concerning why students were not able to (or chose not to) implement innovative strategies that they knew. For example, students may have perceived that standard strategy was what their teacher wanted to see, especially if the standard approach had been the primary focus on instruction (Newton et al., 2010). There may have been cognitive “costs” associated with switching between competing strategies, particularly in terms of longer response times (Luwel et al., 2009; Schillemans et al., 2011). We are currently engaged in studies to address all of these limitations of the present work.

## Author contributions

LX designed the study and wrote the manuscript. RL assisted in the design and implementation of the study, and revised the manuscript. JS assisted in the design and implementation of the study and helped in the writing and editing of the manuscript. JW was in charge of data analysis. YL was responsible for checking the results. RZ assisted in data collection and trained research assistants.

### Conflict of interest statement

The authors declare that the research was conducted in the absence of any commercial or financial relationships that could be construed as a potential conflict of interest.

## Appendix

**Table:** List of equations solved by students during the problem-solving sessions.

**#**	**Problem**	**Standard algorithms**	**Innovative strategies**
1	3(*x* − 1) = 27	3*x* − 3 = 27	*x* − 1 = 9
2	4(*x* + 0.48) = 20	4*x* + 1.92 = 20	*x* + 0.48 = 5
3	$3\left(x+\frac{4}{5}\right)=6$	$3x+\frac{12}{5}=6$	$x+\frac{4}{5}=2$
4	2(*x* + 3) + 3(*x* + 3) = 20	2*x* + 6 + 3*x* + 9 = 20 5*x* + 15 = 20	5(*x* + 3) = 20 *x* + 3 = 4
5	$2\left(x+\frac{4}{7}\right)+3\left(x+\frac{4}{7}\right)=10$	$\begin{array}{cc} 2x+\frac{8}{7}+3x+\frac{12}{7}= & 10\\ 5x+\frac{20}{7}= & 10 \end{array}$	$\begin{array}{cc} 5\left(x+\frac{4}{7}\right)= & 10\\ x+\frac{4}{7}= & 2 \end{array}$
6	4(*x* − 0.29) + 3(*x* − 0.29) = 14	4*x* − 1.16 + 3*x* − 0.87 = 14 7*x* − 2.03 = 14	7(*x* − 0.29) = 14 *x* − 0.29 = 2
7	7(*x* − 2) = 3(*x* − 2) + 16	7*x* − 14 = 3*x* − 6 + 16 7*x* − 3*x* = 14 − 6 + 16	4(*x* − 2) = 16 *x* − 2 = 4
8	$5\left(x-\frac{2}{3}\right)-10=2\left(x-\frac{2}{3}\right)$	$\begin{array}{cc} 5x-\frac{10}{3}-10= & 2x-\frac{4}{3}\\ 5x-2x= & \frac{10}{3}+10-\frac{4}{3} \end{array}$	$\begin{array}{cc} 3\left(x-\frac{2}{3}\right)= & 10\\ x-\frac{2}{3}= & \frac{10}{3} \end{array}$
9	4(*x* + 2.5) + 4*x* = 4(*x* + 2.5) + 8	4*x* + 10 + 4*x* = 4*x* + 10 + 8 4*x* + 4*x* − 4*x* = − 10 + 10 + 8	4*x* = 8
10	$\frac{2x-4}{2}+\frac{6x-12}{3}=3$	$\begin{array}{cc} 6\left(\frac{2x-4}{2}+\frac{6x-12}{3}\right)= & 18\\ 6x-12+12x-24= & 18 \end{array}$	*x* − 2 + 2*x* − 4 = 3
11	$\frac{x+2}{2}+\frac{2x+4}{4}=1$	$\begin{array}{cc} 4\left(\frac{x+2}{2}+\frac{2x+4}{4}\right)= & 4\\ 2x+4+2x+4= & 4 \end{array}$	$\begin{array}{cc} \frac{x+2}{2}+\frac{x+2}{2}= & 1\\ x+2= & 1 \end{array}$
12	$\frac{3x+3}{3}+\frac{4x+4}{4}=4$	$\begin{array}{cc} 12\times \left(\frac{3x+3}{3}+\frac{4x+4}{4}\right)= & 12\times 4\\ 12x+12+12x+12= & 48 \end{array}$	*x* + 1 +*x* + 1 = 4
